# Comparing the Effectiveness of Antidepressants and Cognitive Behavioural Therapy in Preventing Postnatal Depression: A Systematic Review

**DOI:** 10.7759/cureus.92979

**Published:** 2025-09-22

**Authors:** Nadia Shaik

**Affiliations:** 1 Medicine, Salford Royal NHS Foundation Trust, Manchester, GBR

**Keywords:** antidepressants, anxiety, cognitive behavioural therapy, maternal mental health, postnatal depression, ssris, systematic review

## Abstract

Postnatal depression (PND) is a prevalent depressive disorder frequently accompanied by anxiety, with significant implications for maternal health and infant development. This systematic review compared the effectiveness of antidepressant medications and cognitive behavioural therapy (CBT) in the treatment of PND. A systematic search of PubMed and Medline was conducted on November 20, 2019, in accordance with Preferred Reporting Items for Systematic Reviews and Meta-Analyses (PRISMA) guidelines, and randomized controlled trials (RCTs) published between 1997 and 2019 were included. Four RCTs (N = 421) conducted in the UK, USA and Australia met the eligibility criteria. At four weeks, women treated with selective serotonin reuptake inhibitors (SSRIs) demonstrated significantly greater reductions in anxiety symptoms compared to those receiving CBT (95% confidence interval (CI): 1.8-6.5; p < 0.001), with sustained improvement at 18 weeks (95% CI: -3.3 to -0.9; p < 0.001). Conversely, CBT was associated with a faster decline in anxiety symptoms after 12 weeks (p = 0.009). Both interventions achieved significant overall clinical improvements (p < 0.01), while combined therapy did not confer additional benefits beyond monotherapy. These findings suggest that SSRIs may provide superior short-term and sustained relief of anxiety symptoms, whereas CBT may support longer-term symptom control. Therefore, treatment selection should consider symptom severity, potential side effects, breastfeeding and patient preferences.

## Introduction and background

Postnatal depression (PND), also known as postpartum depression (PPD), is a non-psychotic mental health disorder, a complication impacting women usually after childbirth, with a global prevalence of 7%-25% [1]. It has consequences critical to both the mother's and infant's well-being or survival in the long run [2]. Adverse developmental outcomes in children exposed to PND include lower intelligence quotient (IQ), delayed language acquisition and a higher incidence of behavioural difficulties [3,4].

During the initial stages of being a new mother, women go through lifestyle, social, physical, biological and psychological changes, which are believed to begin from the prenatal stage onwards and tend to continue into the puerperium stages [5,6].

While these changes may help some mothers positively cope and adapt to their new lifestyle, they also typically symptomise as leading to irregularity in moods, causing anxiety, depression, postpartum psychosis, “emotional lability, guilt, dysphoria, confusion and even suicidal tendency” [7]. Recent epidemiological evidence suggests that women are twice as likely as men to experience depression during childbearing years [8].

**Figure 1:**
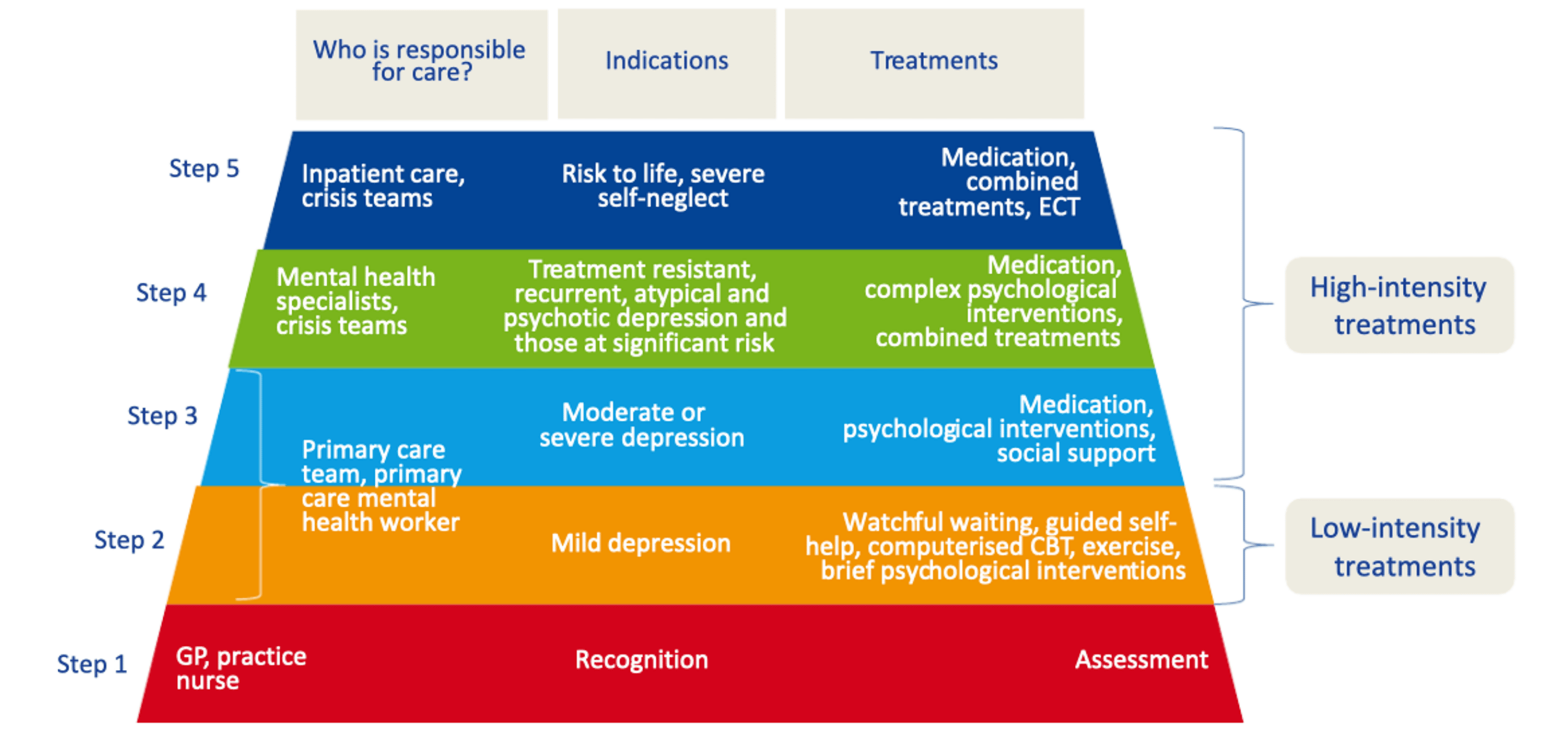
Illustration of the stepped/tiered care approach Source: Derbyshire Healthcare NHS Foundation Trust (2015) [10] CBT: cognitive behavioural therapy, GP: general practitioner, ECT: electroconvulsive therapy

The National Institute for Health and Clinical Excellence (NICE) guidelines on preventative care and multidisciplinary management for PND suggest a tiered care model (Figure 1) involving the fusion of two concepts: “pure stepped care” and “stratified care,” defining a structured approach to increasing treatment intensity associated with providing holistic and patient-centred treatment [9,10].

Public health perspective

Categorised as one of the major global public health problems, nationally, PND affects around 8%-15% of the female population in the UK [11].

Various studies, such as those by Usuda et al. (2016) [12] and Wald et al. (2016) [13], suggest a prevalence rate of 13%-19.2% for PND development within a few months of giving birth, which has encouraged changes in policy by incorporating the use of health visitors in each locality as a supporting service following birth [11]. This service would work alongside general practitioners (GPs) who treat a significant proportion of over 90% of women suffering from PND in a primary care setting.

Additionally, a large-scale study of 700,000 women identified a 13% prevalence of PND, with the associated annual economic burden estimated at approximately £54 million, underscoring the substantial public health and economic impact of the disorder [14]. It also reported statistically higher community care costs for women with PND than for non-depressed women during the postnatal period.

A 2020 meta-analysis combining cohort and case-control studies found that mothers of female infants faced a notably higher risk of postpartum depression [15]. In several countries where male children are culturally preferred, such as India, China, Azerbaijan and Vietnam, research showed increased rates of postpartum depression amongst mothers of girls [16,17]. However, research from Western countries indicated no significant difference in postpartum depressive symptoms related to the sex of the infant [18,19].

The prevalence of PND was also significantly higher amongst women aged 40-44 years, compared to those aged 30-35 years (adjusted odds ratio (aOR): 3.72, 95% confidence interval (CI): 2.15-6.41). Therefore, targeted screening and preventive strategies focused on older postpartum women in the community may be effective in mitigating the elevated risk and associated health burden observed [20].

Clinical perspective

The aetiology of postnatal depression (PND) is influenced by a range of predictive factors, including maternal age, educational level and complications during pregnancy, amongst others (Table 1) [21].

**Table 1:** Predictors for PND Cohen’s d is the standardised effect size, in which 0.2 indicates a small effect, 0.5 indicates a moderate effect and 0.8 indicates a larger effect. Source: O’Hara and Swain (1996) [21] PND: postnatal depression

Predictors for PND	Cohen’s d
Depression	0.75
Anxiety antenatally	0.68
Social support	-0.63
Stressful life events	0.6
Mother’s history of psychopathology	0.57
Self-esteem	-
Childcare stress	-
Neuroticism	0.39
Marital relationship	-0.13
Infant temperament	-
Maternity blues	-
Obstetric variables	0.26
Marital status	-
Negative cognitive attributional style	0.24
Socioeconomic status	-
Unplanned/unwanted pregnancy	-

Identifying risk factors that predispose women to affective disorders during this vulnerable period, prior to illness onset, is essential to enable implementation of effective preventive interventions and reduce the impact on maternal morbidity and mortality rates [22].

Environmental factors such as low socioeconomic status, immigrant background and education level factors are important contributors to PND [23].

Furthermore, a past history of psychological disorders dramatically increases the risk of developing PND. According to NICE (2014), this includes anxiety disorders (13%), psychotic disorders such as bipolar II disorder (22%) with a 70% relapse rate and postpartum psychosis, which demonstrates a 21-fold increase in prevalence, emphasising the critical need for early identification and close monitoring of these high-risk groups. In such circumstances, a referral to a specialist mental health service would be key in preventing further exacerbation of symptoms and, in a worst-case scenario, losing child custody.

A more common phenomenon, termed “baby blues,” affects approximately 30%-80% of women postpartum, characterised by transient feelings of being overwhelmed due to emotional fluctuations [24]. Challenges arise during somatic symptom assessment, as baby blues can cause considerable confusion and are often misinterpreted as requiring treatment rather than being distinguished from PND.

Several screening tools have been developed to facilitate the management of PND, including the Edinburgh Postnatal Depression Scale (EPDS), the Beck Depression Inventory-II (BDI-II) and public health expectancy indicators used internationally. The EPDS is currently the standard clinical tool in the UK, administered in an interview format to identify maternal mood disorders by assessing symptoms such as depressive thoughts and suicidal ideation. Using an EPDS cutoff score of ≥11 optimises the balance between sensitivity and specificity for the detection of postpartum depression [25,26].

Following assessment, a tailored treatment plan can be developed, beginning with the identification of interventions most appropriate for the individual.

Treatment perspective

Treatment categories include pharmacological (antidepressants and anticonvulsants), psychosocial (e.g., peer support and non‐directive counselling) and psychological (e.g., cognitive behavioural therapy and interpersonal psychotherapy) [27]. While these treatments can produce amendable effects on PND symptoms, long-term advantages and disadvantages require specific larger trial evaluations [27].

Cognitive Behavioural Therapy (CBT)

For mild PND, this involves correcting maternal mood by communicating out any “thoughts, perceptions and physical sensations” that often make mothers feel emotionally paralysed and depressed. It focuses on improving the state of mind through daily use of positive coping strategies but is not able to cure any physical symptoms. CBT can be intensified, and the duration for treatment is between three and four months [28].

Antidepressant Medications

Treatment for moderate to severe levels of PND involves using medications that cause chemical changes within the brain for better regulation of emotions and sleeping patterns. This allows for better functioning of the body and alleviates physical symptoms. The typical duration of medication is six months. The efficacy of these medications increases with continual use, even after noticeable improvements can be seen, to avoid relapse of depression. However, a major limitation of antidepressant therapy is that many are not considered safe for use during breastfeeding [28].

Recent evidence from the literature supports the effectiveness of both CBT and pharmacotherapy in the treatment of PND. CBT may provide superior short-term outcomes, whereas pharmacological treatment appears to be more effective for long-term symptom management, as outlined in the Appendices [29].

Lastly, for individuals who do not respond adequately to a single intervention, multimodal therapy may be beneficial. Several studies support the combined use of multiple interventions to provide “adequate therapeutic support,” but no statistical significance in the reduction or remission of symptoms when combining pharmacological and non-pharmacological treatments was identified [29,30].

Aim

The aim of this literature review was to systematically evaluate the effectiveness of cognitive behavioural therapy (CBT) compared with antidepressants in alleviating symptoms and preventing the progression of postnatal depression in mothers.

## Review

Methodology

The primary measure of interest was to compare the effectiveness of CBT and antidepressants in decreasing the progression of symptoms leading to the prevention of PND and its associated effects in mothers. Randomised controlled trials (RCTs) were selected as a study design as they are best suited to evaluate the efficacy of specific interventions.

Stage 1

A literature search was conducted on November 20, 2019, on the EBSCOhost website using Medline. The keywords applied using the advanced search function included “postnatal depression or postpartum depression” AND “CBT or cognitive behavioral therapy” AND “antidepressant medication.” Boolean operators AND/OR were both initially used to explore the research paper options. Further filters were put in place to ensure access to full-text papers written in English only.

Stage 2

Results were refined based on the sensitivity and specificity of papers. Specificity criteria helped in the selection of papers focusing mainly on PND’s effects on the mother, rather than PND’s impacts on maternal parenting or child development. The selected studies were then input into an Excel table (Microsoft Corp., Redmond, WA) for record purposes.

As outlined in Table 2, the framework utilised is a helpful guide for targeted literature searching and establishing review criteria. The inclusion criteria for this literature review were as follows: female participants aged 19-44 years who were mothers, interventions limited to CBT and antidepressants, publication in academic journals reporting only RCTs, articles written in English and publication dates ranging from 1997 to 2019.

**Table 2:** PICO table framework and criteria used PICO: population, intervention(s), comparison(s) and outcomes, CBT: cognitive behavioural therapy

PICO	Criteria
Population	Mothers, setting: community, age: adults (19-44)
Intervention(s)	Behavioural: CBT, medications: antidepressants
Comparison(s)	Behavioural versus medications
Outcomes	Improvement in mental health, quality of life and physical health for mothers
Study design(s)	Randomised controlled trials: for investigating the effects of both interventions on preventing postnatal depression

Exclusion criteria were any studies exploring prenatal stages or effects before the postnatal period. Secondary sources, such as systematic reviews or meta-analyses, were excluded as well.

No restrictions regarding ethnicity, race, sample size or country were established, as PND is a universal phenomenon; hence, results from various countries were allowed for analysis.

Results

Study Selection

**Figure 2:**
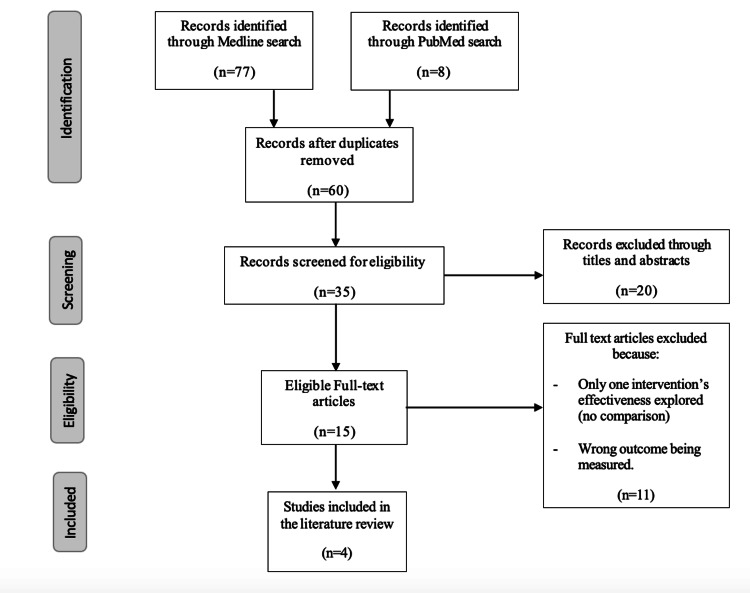
PRISMA flowchart depicting literature search results PRISMA: Preferred Reporting Items for Systematic Reviews and Meta-Analyses

Searches using the databases PubMed Central (PMC), Google Scholar, Scopus and Medline were carried out, using the same search terms mentioned in Methodology, retrieving a total of 85 papers. As outlined in Figure 2, these papers were further screened for eligibility and removal of duplicates, yielding a total of 35 papers.

Full-text articles that were relevant and directly addressed the literature review question were considered, narrowing the list down to 15 papers, out of which only four papers were chosen based on containing adequate results and fulfilling the criteria in accordance with the purpose of the investigation.

Overview of Selected Papers

The four chosen studies (Appleby et al. (1997) [31], Sharp et al. (2010) [32], Misri et al. (2004) [33] and Milgrom et al. (2015) [34]) are outlined in Table 3. All aimed at reporting improvement outcomes of their trial, regarding the reduction in symptoms of PND by evaluating the effectiveness of antidepressants compared to CBT. These studies also had a common secondary aim: investigating whether the use of combined therapy involving both interventions would lead to better efficacy and prevention of PND symptoms.

**Table 3:** Summary of the characteristics of the selected studies CBT: cognitive behavioural therapy, RCT: randomised controlled trial, EPDS: Edinburgh Postnatal Depression Scale, CI: confidence interval, PND: postnatal depression, SSRI: selective serotonin reuptake inhibitor, DSM-IV: Diagnostic and Statistical Manual of Mental Disorders, Fourth Edition, OCD: obsessive-compulsive disorder, BDI-II: Beck Depression Inventory-II

Author and year	Title	Aim/objective	Study design	Participants (country, gender, number)	Intervention/comparison	Results/outcomes	Main conclusions of the paper
Appleby et al. (1997) [31]	A controlled study of fluoxetine and cognitive-behavioural counselling in the treatment of postnatal depression	The aim of this trial was to compare the effectiveness of using fluoxetine (antidepressant) in comparison to CBT and to check for any added benefit of combined treatment using both interventions.	RCT	United Kingdom, N = 87, women	4 comparison groups: fluoxetine and placebo (1 session and 6 sessions), and combinations of drugs + CBT (1 session and 6 sessions), 12-week trial	Significantly greater improvement in EPDS score (95% CI): women receiving fluoxetine over a placebo, fluoxetine = 5.3 (3.7-7.5), placebo = 8.9 (7.1-11.0); women who did 6-week sessions of CBT than 1 week, 1 session = 7.4 (5.7-9.5), 6 sessions = 6.6 (4.6-9.2). No statistical significance was seen in the combined use of both CBT and fluoxetine.	Fluoxetine and CBT are both effective in treating non-psychotic depression (PND). An additional benefit was achieved after the first CBT session by using fluoxetine or additional CBT sessions. Combined therapy shows no additional advantage.
Sharp et al. (2010) [32]	A pragmatic randomised controlled trial to compare antidepressants with a community-based psychosocial intervention for the treatment of women with postnatal depression: the RESPOND trial	The objective of this trial was to evaluate how effective antidepressant treatment is in comparison to psychosocial therapy.	RCT	United Kingdom, N = 254, women (classified criteria 10 for major depression in their first 6 postnatal months)	2 comparison groups: SSRI and non-directive counselling, 18-week trial (after 4 weeks, choice of switching intervention)	Significantly greater improvement in EPDS < 13: women taking antidepressant drugs compared to those in counselling; drugs (4 weeks) = 48/106 (45%), counselling = 20/112 (20%); drugs (18 weeks) = 60/97 (62%), counselling = 56/109 (51%). No statistical significance was seen in efficacy for one treatment over the other.	Antidepressant use illustrates superiority over general supportive care; at 18 weeks, 11% greater improvement was seen in this group. Depending on the preference and circumstances of an individual, a more useful and effective treatment can be chosen. Clinical benefit seen for PND sufferers upon early treatment with antidepressants.
Misri et al. (2004) [33]	The use of paroxetine and cognitive-behavioral therapy in postpartum depression and anxiety: a randomized controlled trial	This study was conducted to investigate any additional benefit of using CBT alongside the typical antidepressant treatment (paroxetine) provided to PND sufferers.	RCT	USA, N = 35, women (DSM-IV diagnosis of PND with comorbid anxiety disorder)	2 comparison groups: monotherapy (n = 16) and paroxetine + 12 sessions of CBT (n = 19), 12-week trial	Significantly greater improvement was seen in women using CBT and paroxetine (p < 0.01). No statistical significance was seen between groups for recovery time or mood/anxiety/OCD.	Receiving antidepressants either through monotherapy or combined therapy had greater efficacy in the reduction of anxiety and depression. No significance was seen in acutely depressed patients when given combined therapy. More research is needed.
Milgrom et al. (2015) [34]	Treatment of postnatal depression with cognitive behavioural therapy, sertraline and combination therapy: a randomised controlled trial	The aim of this trial was to investigate which isolated intervention is more efficacious (monotherapy of CBT versus monotherapy of sertraline) and to investigate the extra benefit of combined therapy versus monotherapy.	RCT	Australia, N = 45, women (DSM-IV diagnosis of depression)	3 comparison groups: CBT, sertraline (SSRI antidepressant) and combined treatment using both, 12-week trial	Significantly greater improvement was seen in the BDI-II score: CBT’s higher decrease in anxiety symptoms (p = 0.009) and CBT producing the lowest depression score for all 5 weeks compared to others: week 2, p < 0.001; week 3, p = 0.02; week 4, p < 0.001; and week 5, p = 0.01.	CBT (monotherapy) is deemed superior in effectiveness due to very fast initial reductions in symptoms compared to antidepressant (monotherapy) and combined therapy. No statistical difference was seen in combined therapy using both sertraline and CBT.

Three studies analysed the sole effectiveness of specific antidepressants (monotherapy) (fluoxetine, paroxetine and sertraline) against a placebo and CBT. Sharp et al. (2010) was the only study using a non-specific antidepressant, compared against a psychosocial therapy [32]. CBT and psychosocial therapy are both classified under psychotherapy; hence, results from this study were viable for comparison.

Overall, the studies involved women of varying sample sizes (35-254 participants), most of whom were chosen 6-8 weeks post-childbirth and often had associated conditions such as anxiety disorder [31]. The Diagnostic and Statistical Manual of Mental Disorders, Fourth Edition (DSM-IV) criteria was a key diagnostic tool used by all four studies to evaluate symptoms of depression and its impact on patients.

Milgrom et al. (2015), amongst others, recognised that the use of antidepressants at early stages of PND had added benefits in improving the outcomes [34]. In contrast, CBT depicted greater longevity in reducing PND symptoms even past the 12-week trial period. Hence, most of the studies appreciated and recognised the time discrepancies at which different interventions were able to produce enhanced improvements.

Key Findings for Outcomes

**Figure 3:**
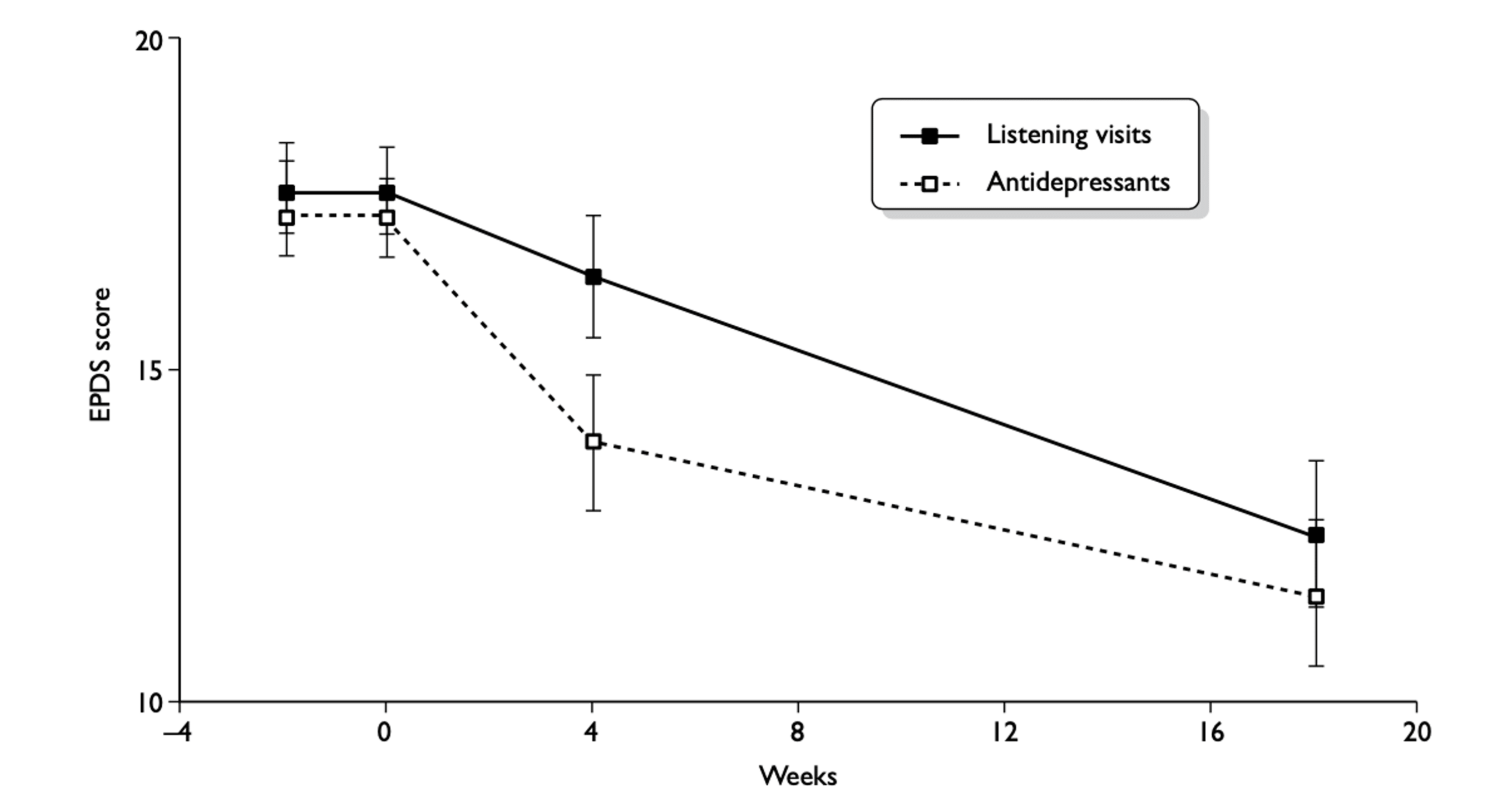
Mean EPDS scores (±2 SE) over time in women randomised to antidepressants or listening visits from baseline to 18-week follow-up Source: Sharp et al. (2010) [32] EPDS: Edinburgh Postnatal Depression Scale, SE: standard error

The use of antidepressants, such as selective serotonin reuptake inhibitors (SSRIs), taken at earlier stages, boosted clinical benefit. As shown by the EPDS continuum in Figure 3, antidepressants demonstrated a statistically significant reduction in symptoms at the four-week follow-up, with a 95% CI of -3.3 to -0.9 (not including 0) and a p-value of <0.001 [32]. Lower scores indicate improved mental health.

Both CBT and antidepressants as monotherapy equivalently provided significant improvement (p < 0.01) in reducing anxiety and mood symptoms [33]. No statistical significance for superiority in efficacy for one group over the other was noted.

Appleby et al. (1997) reported a statistically significant difference between the use of fluoxetine and placebo, with response rates increasing from 37.1% at week 4 to 40.7% at week 12 [31]. Similarly, the study also noted that having six sessions of counselling resulted in a 53.9% response rate at week 4, compared to just 38.7% with a single session by week 12. This illustrates that improvement in health was more probable when participants received treatment over longer periods of time, regardless of the treatment choice.

It can be appreciated that CBT has the ability to provide very fast initial changes in mood symptoms upon treatment commencement. Results from the study by Milgrom et al. (2015) show a high statistical significance (p = 0.008) for CBT in producing a large reduction in individuals categorised as minimally depressed, demonstrating significantly low depression scores compared to other interventions [34].

Combined therapy offered no additional benefit over CBT or antidepressant monotherapies, as all three interventions showed similar symptom reduction over 12 weeks (p < 0.001) [34].

Discussion

Interpretations

Preferred outcomes regarding effective reduction in anxiety and mood symptoms for this literature review were shown in no favour for a particular intervention, as both antidepressants and CBT interventions demonstrated evidence for equivalent positive improvement in health and symptoms for PND sufferers.

The findings from Sharp et al. (2010) suggest that antidepressants play a pivotal role in a patient’s course of therapy due to their effectiveness in being applicable for both short-term and long-term use. Long-term use of medications such as SSRIs for at least six months provides strong evidence for reducing relapse rates [32]. However, it is critical to perform frequent observations of neonates for mothers breastfeeding while taking these medications. Short-term use of SSRIs is considered a first-line treatment choice due to their greater clinical benefit when initiated early.

The above key findings depict that by the 12th week, the efficacy for both interventions was significantly similar. Therefore, it can be concluded that an effective therapy designed for PND sufferers can initially begin with antidepressants within the first four weeks, later to be followed with CBT sessions, as results showed high CBT efficacy in the latter second stage of the trial.

Misri et al. (2004) shared similar attitudes regarding combined therapy as it was less cost-effective and had no additional benefit in relieving PND symptoms in acutely depressed patients [33].

Milgrom et al. (2015) was the only study whose findings did not appear consistent with the other three research papers [34]. It presented results showing CBT’s superiority in efficacy and detectable statistical differences upon use, compared to sertraline and combined therapy. However, it was in support of other studies regarding the futile use of combined therapy for short term. The variation in results may have originated from the differences in methods for screening participants, impacting the quality of the results. Also, this trial only used a specific antidepressant drug, sertraline; the efficacy of this drug for that particular level of depression may differ in comparison to taking paroxetine and fluoxetine, which has not been explored.

On the contrary, Appleby et al. (1997) depicted greater improvement in participants’ health after one week of taking fluoxetine, illustrating beneficial effects in early use of antidepressant [31]. Interestingly, similar levels of improvement were seen in participants who were going to receive the treatment in the future but had not. The trial highlighted the importance of CBT counselling, which goes hand in hand with medications. Perceived appraisal of the situation, as well as physical and emotional support, was beneficial in improving health outcomes.

Similarly, the findings by Milgrom et al. (2015) suggested that CBT may have the potential to produce rapid initial improvements in mood [34], although these effects were not consistently observed across all four trials, likely due to variations in compliance and treatment adherence, which may also account for the limited incremental benefit observed with combination therapies, as they are also hard to comply with.

Limitations and Bias

The prime limitation of the trial by Sharp et al. (2010) was that it allowed women after four weeks to switch interventions due to its pragmatic design; this led to difficulty in figuring out which intervention had provided the most improvement in health [32].

Current research on pharmacological treatments for PND predominantly focuses on SSRIs; this narrow scope limits the generalisability of findings to other classes of antidepressants, such as serotonin-norepinephrine reuptake inhibitors (SNRIs), tricyclic antidepressants (TCAs), or atypical agents, which may also have therapeutic potential in this population.

The study conducted by Misri et al. (2004) included a sample size of only 35 women, which was too small [33]. Variation in sample size was also one of the major limitations of the present study, especially when a large number of eligible women declined participation [34]. The relevance of the sample size is key for the generalisation of results because a sample that is too small may not be a wider representation of the true population.

Loss to follow-up limited the trials conducted, as a large number of participants failed to complete treatment or to follow up on the questionnaire, adding further barriers in forming conclusive results; only 61 (70%) women completed 12 weeks of treatment, as discussed in the study by Appleby et al. (1997) [31].

Studies that indicated self-assessment of participants using scheduled clinical interviews as a psychometric measure or treatment compliance checklists could have created bias within the results. In addition to this, Sharp et al. (2010) noted that participants who volunteered for the trial could be presumed to be more accepting of the treatment [32]. Therefore, the need for trials representing a likely scenario of a woman to be seen by a health service that routinely screens and treats PND should be considered.

The included studies had several strengths as well. Appleby et al. (1997) utilised randomisation and blind trials as methods for tackling bias, by assigning treatments that were selected at random in a 1:1:1 ratio [31]. In addition to this, a selection of papers used different validated psychometric measures for evaluating cognitive-affective and physiological symptoms of depression. These included the Beck Depression Inventory-II (BDI-II) in Milgrom et al. (2015) [34] and the Edinburgh Postnatal Depression Scale (EPDS) in Sharp et al. (2010) [32], which improved the validity and reliability of diagnosis in the studies.

## Conclusions

In summary, both CBT and antidepressants are equally effective in reducing PND in women. However, the findings of this literature review depict that the choice of interventions depends on the intensity of the depression. CBT is appropriate for mild to moderate episodes, whereas antidepressants may come in use for severe episodes of PND. Therefore, the decision for the selection of treatment is based on an individual’s circumstances and preferences, as both interventions would be suitable for effectively maintaining symptoms and reducing progression of PND.

Future research should be considered in carrying out newer trials exploring the efficacy of combined therapy, in order to provide better options for patients who are not necessarily responsive to monotherapy interventions.

## Appendices

Table 4 summarizes the stages of PND, their clinical features and risks, and recommended interventions.

**Table 4:** Stages of postnatal depression and suggested interventions Source: Fitelson et al. (2010) [35] PND: postnatal day, PPD: postpartum depression, EPDS: Edinburgh Postnatal Depression Scale, CBT: cognitive behavioural therapy, IPT: interpersonal psychotherapy, SSRI: selective serotonin reuptake inhibitor

Stage	Clinical features	Risks	Recommended interventions [35]
Early postpartum (PND 1-14): adjustment period	Emotional lability, tearfulness, irritability, “baby blues” common and self-limiting	Progression to PPD if symptoms persist beyond 2 weeks	Psychoeducation for mother/family, early screening (e.g., EPDS), social and partner support, adequate rest and lactation support
Acute postnatal depression (PND 2-12 weeks)	Persistent sadness, anhedonia, guilt, impaired bonding, appetite/sleep disturbances	Impaired maternal-infant interaction, escalation to severe depression	First-line psychological therapies (CBT and IPT), peer/mother-infant therapy; if moderate-severe, SSRIs (e.g., sertraline for breastfeeding)
Persistent postnatal depression (PND 3-6 months)	Ongoing depressive symptoms, functional impairment, comorbid anxiety common	Negative impact on infant emotional regulation, attachment and cognitive development	Combination treatment psychotherapy + antidepressants, hormone-based therapies, parenting interventions, structured home visiting programs
Chronic postnatal depression (PND 6-12+ months)	Depression persists beyond 6 months; may evolve into recurrent major depression	Long-term maternal mental health burden	Long-term psychiatric care medication + therapy; relapse prevention strategies, family therapy, infant early intervention programs (attachment-based, developmental monitoring)
